# Software-Assisted Data Processing Workflow for Intact Glycoprotein Mass Spectrometry

**DOI:** 10.1021/acs.jproteome.2c00762

**Published:** 2023-03-01

**Authors:** Alan B. Moran, Elena Domínguez-Vega, Manfred Wuhrer, Guinevere S. M. Lageveen-Kammeijer

**Affiliations:** † Center for Proteomics and Metabolomics, Leiden University Medical Center, 2300 RC Leiden, The Netherlands; ‡ Department of Analytical Biochemistry, Groningen Research Institute of Pharmacy, University of Groningen, 9713 AV Groningen, The Netherlands

**Keywords:** prostate-specific antigen, proteoforms, intact protein, mass spectrometry, data processing, deconvolution

## Abstract

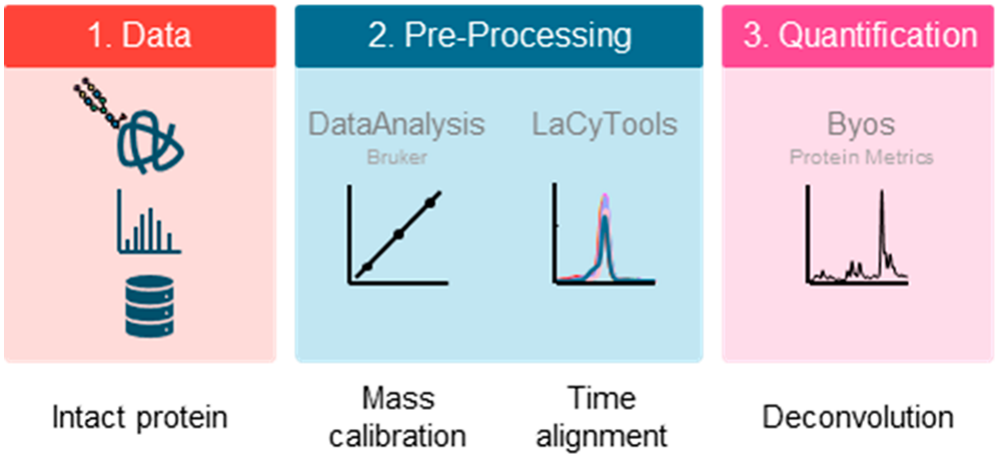

Intact protein analysis by mass spectrometry is important for several applications such as assessing post-translational modifications and biotransformation. In particular, intact protein analysis allows the detection of proteoforms that are commonly missed by other approaches such as proteolytic digestion followed by bottom-up analysis. Two quantification methods are mainly used for intact protein data quantification, namely the extracted ion and deconvolution approaches. However, a consensus with regard to a single best practice for intact protein data processing is lacking. Furthermore, many data processing tools are not fit-for-purpose and, as a result, the analysis of intact proteins is laborious and lacks the throughput required to be implemented for the analysis of clinical cohorts. Therefore, in this study, we investigated the application of a software-assisted data analysis and processing workflow in order to streamline intact protein integration, annotation, and quantification via deconvolution. In addition, the assessment of orthogonal data sets generated via middle-up and bottom-up analysis enabled the cross-validation of cleavage proteoform assignments present in seminal prostate-specific antigen (PSA). Furthermore, deconvolution quantification of PSA from patients’ urine revealed results that were comparable with manually performed quantification based on extracted ion electropherograms. Overall, the presented workflow allows fast and efficient processing of intact protein data. The raw data is available on MassIVE using the identifier MSV000086699.

## Introduction

The analysis of intact proteins by mass spectrometry (MS) involves the examination of the complete protein, including various post- and cotranslational modifications.^1^ During electrospray ionization (ESI)-MS, a protein may be analyzed under native or denaturing conditions.^2^ The latter is referred to here as intact protein analysis, and involves the application of volatile MS-compatible solvents with a low pH in order to improve the solubility and ionization of the protein.^2^ Furthermore, an online separation technique is often employed prior to the introduction of the protein into the MS. For example, reversed-phase liquid chromatography (RPLC)-MS is commonly employed to analyze biotherapeutics,^3,4^ yet this approach has been reported to show poor peak resolution of proteoforms during intact protein analysis.^1^ In contrast, hydrophilic interaction LC (HILIC)-MS has demonstrated efficient separation of protein glycoforms.^5,6^ In addition, capillary electrophoresis (CE)-ESI-MS has been recognized as an excellent technique to investigate intact proteins as proteoforms may be separated based on their intrinsic properties, including also post-translational modifications.^7,8^

Intact protein analysis offers multiple advantages over solely performing a protease digestion and bottom-up investigation. For example, minimal sample preparation is required, thus there is a smaller likelihood that modifications may be introduced to the protein and less time is needed for sample processing. In addition, different proteoforms of the protein may also be observed during the analysis.^9^ Despite this, a global approach that incorporates information from different levels, from top-down to bottom-up, is often required during protein analysis^2^ and enables the analysis of the entire protein as well as cross-validation between the results.^10^

When MS is hyphenated with a separation technique, there are two predominant methods of performing data processing and quantification of intact protein spectra, namely either via extracted ion chromatogram (XIC) or mass deconvolution approaches. Both techniques have been well covered in recent reviews.^1,11,12^ Briefly, the XIC method involves the determination of the area under the peak via selection of one or several *m*/*z* that generally cover the most abundant charge states of the protein. In order to maximize sensitivity and specificity, the selection of the charge states as well as the width of the mass window are important parameters for this method, respectively.^13^ In comparison, deconvolution employs an algorithm to convert the multiple charge states observed in an intact protein mass spectrum into a neutral spectrum that demonstrates the masses of the observed proteoforms. There are several algorithms available to perform this function with maximum entropy^14^ being the most commonly employed by most data processing softwares,^15^ although there is the emergence of more recently developed approaches such as parsimonious charge deconvolution.^15^ Importantly, the input *m*/*z* range as well as the output range require optimization by the user to ensure a suitable number of charge states of the protein are included in the formula, while also aiming to reduce the production of any artifacts due to the data processing algorithm.^1,11,15−17^ Overall, there is still no clear consensus in the field as to which is the most suitable technique to apply when performing intact protein data processing.^1,17^ Undoubtedly, in order to develop a set of best data processing practices, there is a need for comparisons between the two approaches to be made within the same software, as well as software offered by different vendors.^13,17^

In general, several intact protein studies are mainly concerned with the absolute quantitation of biotherapeutics,^13,16−19^ although the determination of proteoform relative abundance has also been applied for the quantification of drug-antibody ratios.^20,21^ Despite this, the determination of best practices for intact protein data processing, based on these studies, does not encompass challenges faced in a biomarker discovery setting. For example, relative quantification is a suitable approach for the quantification of intact proteoforms in the clinical setting as differences between patient groups may be readily observed.^1^ Furthermore, the approaches applied for assessing biotherapeutics^16−19,22,23^ may not account for patient to patient variation that is observed in clinical assays.^7^ Finally, it has been recognized that the throughput of data processing is one of the main challenges facing the intact protein analysis of clinical cohorts whereby large numbers of samples are required to derive statistically significant data.^7^

In this study, we sought to improve the throughput and efficiency of intact protein data processing by developing a software-assisted workflow. This approach was demonstrated using urinary PSA to compare the deconvolution (software-assisted) quantification results with the previously published extracted ion electropherogram (XIE) data (manual),^7^ both of which were generated by using two different software tools. In addition, we also further examined the proteoform profile of prostate-specific antigen (PSA) by performing orthogonal analyses of seminal PSA via intact protein, middle-up and bottom-up approaches, and compared this with the previously established profile of urinary PSA.^7^

## Experimental Section

### Sample Preparation

The sample preparation of urinary PSA, including sample collection, immunocapturing, and in-solution tryptic digestion, has previously been described.^7,24^ Seminal PSA standard (Lee BioSolutions, St. Louis, MO) was prepared for intact protein analysis as follows: PSA was reconstituted (2.2 μg/μL) in LC-MS grade H_2_O (Fluka, Steinheim, Germany) and buffer-exchange was carried out using centrifugal filters with a 10 kDa MWCO (Merck Life Science, Amsterdam, The Netherlands). This was performed by conditioning the filter with 500 μL of H_2_O followed by centrifugation (14,000*g* × 5 min). The filtrate was discarded and the sample (26 μg) was added to the filter. The volume was made up to 500 μL in total with H_2_O. Another centrifugation step was performed and the filtrate was discarded. Then, 250 μL of H_2_O was added, centrifugation was carried out (14,000*g* × 5 min), and the filtrate was removed. This was repeated three times in total. Finally, the sample was retrieved by inverting the filter into a fresh tube and centrifuging (4000*g* × 5 min).

The reduction (and alkylation) of seminal PSA for middle-up analysis was carried out with PSA prepared at a concentration of 2.2 μg/μL. The sample (100 μg) was added up to 100 μL with H_2_O in an Eppendorf tube (1.5 mL). Then, 1 μL of 200 mM DL-dithiothreitol (DTT, Sigma-Aldrich, Steinheim, Germany) was added at a final concentration of 2 mM. The sample was vortexed for one min and heated at 60 °C for 30 min. Following this, 1.5 μL of 400 mM iodoacetamide (IAA, Sigma-Aldrich) was added (final concentration of 6 mM). For the preparation of reduced samples without alkylation, the same volume of H_2_O was added instead of IAA. The samples were incubated at room temperature (RT) in the dark for 60 min. DTT (200 mM) was added (3 μL) at a final concentration of 6 mM. This was followed by an incubation at RT in brightness for 20 min. Finally, the samples were desalted by performing the buffer-exchange procedure as described above.

### CE-ESI-MS

The CE experiments were carried out using a CESI 8000 (Sciex, Brea, CA). All capillaries were sheathless bare-fused silica (BFS) with a porous tip (91 cm, 30 μm i.d. × 150 μm o.d.) and in the case of intact protein and middle-up analysis, capillaries were coated in-house with polyethylenimine (Gelest, Morrisville, NC)^25^ as previously published.^7^ Prior to the separation, background electrolyte (BGE) consisting of 20% glacial acetic acid (HAc, Sigma-Aldrich) was prepared (v/v, 3.49 M, pH 2.3) and was used to rinse (100 psi × 5 min) the separation line. Then, the conductive capillary was filled (100 psi × 4 min) with BGE and the sample was hydrodynamically injected. In the case of seminal PSA, an injection of 2.5 psi × 15 s was applied (approximately 5 nL, 0.8% of the total capillary volume). Finally, separation was achieved by applying −20 kV over 45 min with the capillary temperature set to 15 °C.

As previously published,^7,24^ the CE separation of PSA tryptic peptides for bottom-up was performed on noncoated BFS capillaries which were conditioned by applying 0.1 M NaOH × 2.5 min, then H_2_O × 3 min, 0.1 M HCl × 2.5 min and H_2_O × 3 min. Following this, the BGE was applied for 3 min. The digested seminal PSA standard was prepared at a concentration of 100 ng/μL, and 6.7 μL was mixed with 3.4 μL of the leading electrolyte, 1.2 M ammonium acetate, pH 3.39 (Fluka). Hydrodynamic injection was performed (1 psi × 60 s), corresponding to a volume of 8 nL (1.3% capillary volume). Then, an injection (0.5 psi × 25 s) of a BGE post plug was carried out. Following this, a separation voltage of 20 kV was performed for 80 min at 15 °C.

The CESI 8000 was coupled with a maXis Impact Ultra-High Resolution QqTOF MS (Bruker Daltonics GmbH, Bremen, Germany) equipped with a nanoelectrospray source. The MS acquisition parameters were previously published for the intact protein and middle-up approaches^7^ as well as bottom-up analysis.^24,26^ Importantly, in order to perform fragmentation of small peptides generated via internal cleavage of the protein followed by tryptic digestion, the following parameters were applied for a bottom-up approach using a concentrated sample of digested seminal PSA standard (100 ng/μL): electrospray voltage, 1250 V; nitrogen drying gas, 1.2 L/min at 150 °C; quadrupole ion energy, 3 eV; collision cell energy, 7 eV; transfer time, 130 μs; prepulse storage time, 15 μs; *m*/*z* range, *m*/*z* 150–3500.

### Data Processing

Seminal PSA data, generated by intact protein, middle-up, and bottom-up analysis, was directly analyzed using Byos (v4.4, Protein Metrics, Cupertino, CA) in the Bruker DataAnalysis file format (.d). In addition, a software-assisted data processing workflow was applied to the urinary PSA intra- and interday (*n* = 9), and patient (*n* = 8) data sets. The workflow consisted mainly of three stages, first a 12-point internal mass calibration was performed in Bruker DataAnalysis (v5.0) using the *m*/*z* of the most abundant nine charge states from the most abundant PSA proteoform, active PSA containing H5N4F1S2 (28430.91 Da; 2187.9913^13+^, 2031.7782^14+^, 1896.3934^15+^, 1777.9318^16+^, 1673.4068^17+^, 1580.4958^18+^, 1497.3648^19+^, 1422.5469^20+^, 1354.8545^21+^). In addition, the *m*/*z* of internally spiked PSA (LSEPAELTEAVK; 1286.6837^1+^, 643.8454^2+^) and IgG (GPSVFPVAPSSK; 1172.6309^1+^) peptides (developed in-house by FMoc solid phase peptide synthesis) were also used in the mass calibration. Second, the data was converted to .mzXML format and migration time alignment was performed in LaCyTools (v2.01)^27^ using abundant *m*/*z* values found in each sample (Supporting Information, Table S1). Finally, the aligned .mzXML data files were imported into Byos (v4.4) by Protein Metrics and the base peak electropherogram trace was used for automatic integration, annotation, and quantification via deconvolution. The version of Byos in this work included a beta-release feature of the mass XIC function for visualization of XIC data. This feature has subsequently been officially released in Byos v4.5.

The parameters for manually generating and integrating XIEs using Bruker DataAnalysis (v5.0) were reported previously whereby the three most abundant charge states with an extraction window of ± *m*/*z* 0.1 were combined to generate an XIE (Gaussian smoothing, 2 points) for each proteoform.^7^ In the current study, the following deconvolution settings were applied to intact protein and middle-up data: charge vector spacing, 0.6; smoothing sigma *m/z,* 0.02; spacing *m*/*z*, 0.04; mass smoothing sigma, 3; mass spacing, 0.5; minimum charge, 5; iteration maximum, 10. In the case of spectra with isotopic resolution, such as some of the fragments observed in the middle-up data, the following deconvolution parameters were used instead: charge vector spacing, 0.5; smoothing sigma *m/z,* 0.01; spacing *m*/*z*, 0.01; mass smoothing sigma, 0.1; mass spacing, 0.1; minimum charge, 3; iteration maximum, 10. In addition, a *m*/*z* input range/mass output range of *m*/*z* 1000–3000/26000–30000 Da and *m*/*z* 600–3000/1000–30000 Da was applied for the intact protein and middle-up data, respectively. However, the input and output ranges for the middle-up deconvolution settings were also further modified per peak to enable the search for fragments of different sizes and abundances. Furthermore, the integration windows used for the generation of deconvoluted spectra in urinary PSA may be found in Supporting Information, Table S2. In addition, bottom-up data was examined using the following processing parameters: minimum MS2 score, 15; maximum precursor *m*/*z* error, ± 20 ppm; maximum fragment *m*/*z* error, ± 20 ppm; missed cleavages, 2; fixed modification, carbamidomethyl. Importantly, fully specific and N- and C-term ragged searches were applied in order to search for peptides that have amino acid loss due to naturally occurring internal cleavage of the protein.

### Intact Proteoform Assignments

In order to automatically annotate the observed masses in the deconvoluted spectra, a delta mass list including glycan masses previously determined by MS/MS using a bottom-up approach^24^ as well as expected cleavage variants and internal amino acid loss,^7^ was generated (Supporting Information, Table S3). In the case of mass calibrated data a mass error cutoff was applied (±25 ppm). Furthermore, annotations were only considered if they followed the expected order of migration as shown in Supporting Information, Table S2. In addition, cleavage sites were specified if corresponding fragments and peptides were found in the middle-up and bottom-up data sets, respectively (Supporting Information, Table S4). Notably, the annotation of fragments in the middle-up results was further supported by the comparison of the reduced versus reduced and alkylated masses. Importantly, the mass was expected to increase by the corresponding number of cysteines (+57.05 Da per cysteine) that were present in each fragment due to the alkylation step.

## Results and Discussion

The current study further characterizes cleaved proteoforms and glycoforms in seminal PSA via CE-ESI-MS by assessing orthogonal data generated by intact protein, middle-up, and bottom-up approaches. These assignments were then compared with the previously established profile of urinary PSA.^7^ Furthermore, the software-assisted workflow for intact protein data processing was developed by implementing three software tools to perform mass calibration (DataAnalysis), migration time alignment (LaCyTools), and deconvolution quantification (Byos). Finally, the developed workflow was applied in order to analyze intact urinary PSA proteoforms, incorporating new assignment information from the aforementioned orthogonal data sets, and performing a comparison with previously published quantification results.^7^

### Orthogonal Data Analysis of Seminal PSA

**Figure 1:**
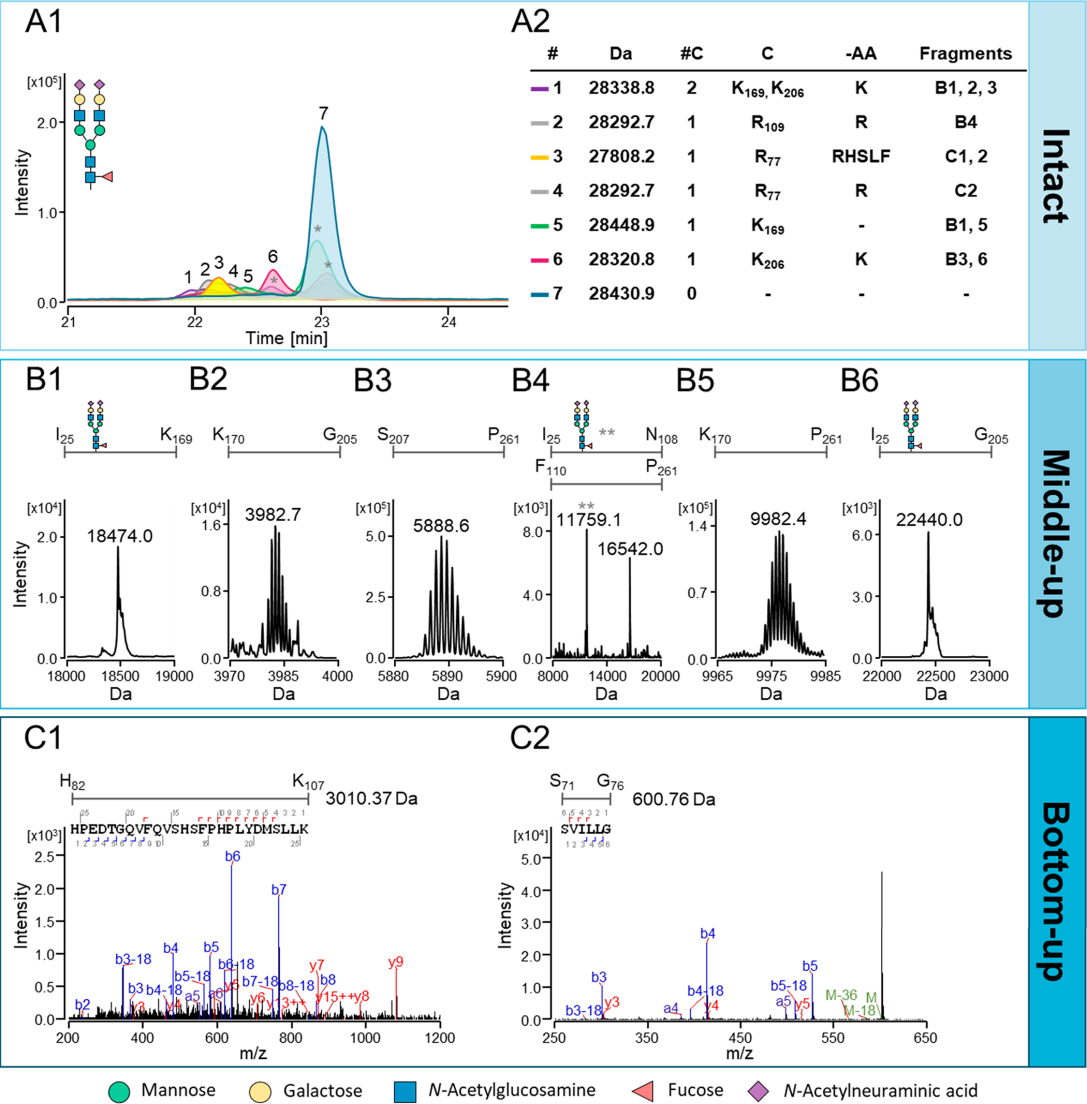
Analysis of seminal PSA via orthogonal approaches; (A) intact protein, (B) middle-up, and (C) bottom-up. (A1) Electrophoretic profile of seminal PSA with XIE peaks 1–7. Only proteoforms with the most abundant glycoform H5N4F1S2 are shown. Asterisk (*) denotes XIEs from overlapping *m*/*z* that are present in the charge envelopes of several different proteoforms. The table in (A2) shows the underlying proteoforms including the peak number (#), intact mass (Da), number of cleavages (#C), cleavage site (C), amino acid loss (-AA), and masses (Fragments) that support the assignment from the middle- and bottom-up approaches. PSA fragments found during middle-up analysis are shown in (B1–B6) whereby the first and last residue of the fragment as well as the glycoform are represented above the deconvoluted spectra. The average mass is illustrated except when isotopic resolution is achieved, in which case the mass of the most abundant isotope is demonstrated. In B4, two fragments are shown, I_25_–N_108_, containing H5N4F1S2 (11759.1 Da) as illustrated by the double asterisk (**), and F_110_–P_261_ (16542.0 Da). Two PSA tryptic peptides that were found as a result of prior internal cleavage of the protein at and loss of R_77_ are shown (C1 and C2).

In Figure 1.A1, seminal PSA proteoforms with cleavages at various cleavage sites migrate first, as shown by peaks 1–6. Notably, the cleaved proteoforms with the most abundant glycoform, H5N4F1S2, are shown in Figure 1.A1. This is then followed by noncleaved PSA whereby these proteoforms migrate in order of decreasing sialic acid content, from tri- to nonsialylated (Supporting Information, Figure S1). The electrophoretic profile of intact seminal PSA was similar to the urinary PSA profile as previously demonstrated.^7^ The cleavage site and number of cleavages of PSA proteoforms are determined based on the amino acid loss from the internal sequence and the total number of water molecule (+18 Da) additions, respectively.^7^ The intact mass gives the sum of all modifications to the protein and, therefore, it is useful to further dissect the nature and site of modifications via orthogonal approaches to support intact protein assignments.

PSA with double-cleavage was observed migrating in peak 1 (22.0 min) in Figure 1.A1. The mass 28338.8 Da was tentatively assigned as having cleavages at K_169_ and K_206_, and the loss of one K residue. In the previous study with urinary PSA, the proteoform with the mass 28338.8 Da was assigned as double-cleavage variant at the site E_145._^7^ However, this assignment is revised in the current study based on new evidence. For example, cleavage at K_169_ and K_206_ is supported by the fragments B1–3 in Figure 1 and, importantly, fragment B2 (3982.7 Da) is the result of a double cleavage at K_169_ and K_206_, and the loss of K_206_. In addition, the peptide V_138_–F_165_ with amino acid loss of LTPK_169_ was observed in the bottom-up analysis (Supporting Information, Table S4). Finally, the loss of a positively charged K decreases the net positive charge of the proteoform which is expected to decrease the migration time of cleaved PSA. In contrast, the loss of a negatively charged E would increase the net positive charge of the protein, thus increasing its migration time. Moreover, no evidence for fragments or peptides associated with cleavage at E_145_ could be found by the middle-up or bottom-up approaches. Overall, these results suggest that that the double cleavage proteoforms observed in seminal and urinary PSA are due to cleavages at K_169_ and K_206_. Significantly, PSA with a cleavage at K_169_ and K_206_ is also referred to as benign PSA (bPSA) due to its association with the development of Benign Prostate Hyperplasia (BPH).^28,29^ Thus, the revision of this assignment as a result of new evidence offered by orthogonal approaches enables this intact PSA assay to be used to identify and monitor the abundance of bPSA.

Two single-cleavage isoforms with the loss of R (28292.7 Da) were detected at peaks 2 and 4 in Figure 1.A1. In addition, cleaved PSA at R_77_ and loss of RHSLF (27808.2 Da) was observed in peak 3 in the intact protein profile. Further investigation by middle-up analysis detected the fragments I_25_–G_76_, H5N4F1S2 (8184.0 Da) and H_78_–P_261_ (20689.4 Da) which correspond to cleavage at and loss of R_77_. In addition, reduced seminal PSA showed that the fragment H_82_–P_261_ with the loss of HSLF_81_ (20204.9 Da) migrated earlier than H_78_–P_261_, most likely due to the loss of the positively charged H residue (Supporting Information, Table S4 and Figure S2). This was reflected in the intact protein profile as 27808.2 Da (peak 3) migrates before 28292.7 Da (peak 4), corresponding to cleavage at the site R_77_, with and without loss of HSLF, respectively. Cleavage at this site is also supported by bottom-up analysis as peptides with loss of HSLF_81_ (C1) and R_77_ (C2) were found, next to nontruncated forms (Supporting Information, Figure S3). Additionally, a 10.7 kDa fragment was observed in the seminal PSA standard which may correspond to the fragment S_79_–V_174_ (Supporting Information, Table S5). This fragment may also be due to cleavage at R_77_, although sequence confirmation is required in order to confirm this as other peptides within the PSA sequence, such as T_150_–Y_249_, also correspond to this mass. Interestingly, the 10.7 kDa fragment was not found in urinary PSA^7^ and it should be further explored whether this fragment is specific to seminal PSA and whether it is a degradation product following cleavage at R_77_. Importantly, this is the first study that reports on a cleavage at R_77_ of PSA in any matrix.

Fragments were also found by middle-up analysis that demonstrate cleavage at R_109_. For example, the fragments found in Figure 1.B4 correspond to cleavage at this site with loss of R (Supporting Information, Table S4). Thus, it may be determined that the mass of 28292.7 Da in peak 2 belongs to the PSA proteoform with a cleavage at R_109_. The mass 28292.7 Da is also observed in urinary PSA^7^ and based on the relative migration time (relative to the most abundant peak), likely the R_109_ cleavage variant is also observed in urinary PSA. Despite this, the relative migration times reported in Supporting Information, Table S4 show some variation and due to the close migration times of the 28292.7 Da isomers in seminal PSA, further investigation by a middle-up approach is required for urinary PSA in order to confirm whether this proteoform is due to cleavage at R_109_ or R_77_.

The single cleavage PSA variant with no amino acid loss (28448.9 Da) was detected under peak 5 in Figure 1.A1. The cleavage site may not be determined in the intact profile due to the absence of any amino acid loss. However, the middle-up fragments B1 and B5 illustrate that cleavage occurs at K_169_ and this is the only cleavage site whereby no amino acid loss is observed in the middle-up analysis. Thus, the mass 28448.9 Da may be inferred as having a cleavage at the K_169_ site. However, no tryptic peptides for this cleavage site could be found by the bottom-up approach as K is also the cleavage site targeted by trypsin. Thus, further investigations should examine this by generating peptides with alternate enzymes such as Arg-C.

**Scheme 1:**
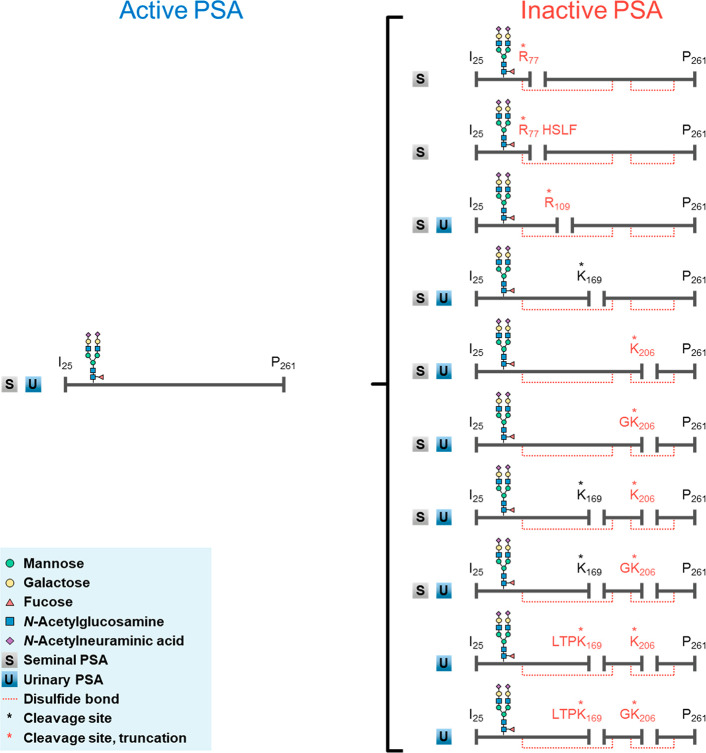
Overview of the Cleaved Proteoforms Found in Seminal and Urinary PSA Noncleaved, active PSA undergoes internal cleavage which inactivates the protein. Cleaved PSA proteoforms arise at one (single cleavage) or two (double cleavage) of four cleavage sites, as well as further truncated variants. Notably, PSA contains five disulfide bonds in total whereas the red dotted lines are shown here to represent how the overall structure of the protein is kept intact following cleavage via the disulfide bonds. Ten cleaved proteoforms are observed in total across seminal and urinary PSA, in addition to the glycoforms for each proteoform (Supporting Information Tables S6 and S7). Only the most abundant glycoform, H5N4F1S2, is illustrated here. The legend may be found in the blue box.

Peak 6 (22.7 min) in Figure 1.A1 shows PSA with a single cleavage and loss of K (28320.8 Da), likely due to cleavage at K_206_. In addition, the mass 28263.7 Da also elutes at 22.7 min and is assigned as a cleavage at K_206_ with the loss of GK, as it is expected that further loss of a noncharged amino acid at the same cleavage site would not result in any shift to the migration time of this proteoform (Supporting Information, Table S4). Furthermore, cleavage at K_206_ is also supported by new evidence provided by the middle-up approach. The fragments B3 (5888.6 Da) and B6 (22440.0 Da) are shown in Figure 1, which correspond to PSA with cleavage at K_206_ and loss of K. Furthermore, the tryptic peptide WTGG_205_ was found (Supporting Information, Table S4 and Figure S3) which contains the loss of K_206_. Although the expected fragment or peptide (I_25_···G_204_; H5N4F1S2 and WTG_204_, respectively) containing the loss of GK was not observed, Supporting Information, Table S4 shows that a fragment arising due to double-cleavage (K_170_···G_204_) was observed that contains the loss of GK_206_. To summarize, Scheme 1 shows that PSA contains a fascinating diversity of proteoforms, which includes noncleaved (active) and cleaved (inactive)^30^ PSA found in seminal and urinary PSA during this study.

### Data Processing of Intact Proteoforms

In the previous study, we performed a “manual” approach for quantification using the DataAnalysis software. In this case, the large charge envelope of the intact proteoforms resulted in overlapping *m*/*z* signals and broad XIE peaks. Thus, it was necessary to manually integrate and deconvolute each XIE peak in order to determine peak areas and masses, respectively. In the current study, we present a workflow primarily using Byos in order to perform “software-assisted” intact protein data processing and deconvoluted quantification of peak intensities. In order to perform the comparison, both approaches were used to asses two data sets: an intra- (*n* = 3) and interday (*n* = 9) study which demonstrated the intermediate precision and repeatability of the intact urinary PSA assay using a patient urinary pool, and the measurement of individual patient samples (*n* = 8).^7^ Notably, the proteoforms for several intact masses determined in intact urinary PSA are revised in Supporting Information, Table S4 based on the evidence obtained from the orthogonal analysis of seminal PSA.

In Table 1, the annotation of proteoforms in the intra- and interday data set shows that implementing the software-assisted workflow in the current study resulted in the assignment of 35 proteoforms, including nine unique masses that were not observed when manual processing was performed. However, these unique masses were mainly very low abundant proteoforms with relative abundances <1% (Supporting Information, Table S6). In comparison, manual processing determined 32 proteoforms, including six unique masses. Additionally, 26 proteoforms were detected by both methods. The analysis of the individual patient data showed that 23 proteoforms were quantified in total by both data processing techniques. However, four unique proteoforms were determined each by the software-assisted and the manual approach, respectively, and 19 masses were quantified by both approaches.

**Table 1:** Comparison of Mass Assignments and Quantification between the Software-Assisted (SA) Workflow and the Manual Approach for the Intra- and Interday, and Patient Data Sets^a^

		Mass Assignments			Quantification (RSD)			
Data Set	Method	Total	Unique	Common	Intraday 1	Intraday 2	Intraday 3	Interday
Intra and interday	SA	35	9	26	13%	9%	12%	19%
	Manual	32	6		12%	8%	12%	15%
Patients	SA	23	4	19	n/a	n/a	n/a	n/a
	Manual	23	4		n/a	n/a	n/a	n/a

a Mass assignments refers to assignment of proteoforms to deconvoluted masses that were found within the mass error threshold (±25 ppm), as well as demonstrating an expected migration time. “Unique” masses are proteoform assignments that were only determined by one data processing method whereas “common” refers to proteoforms determined by both techniques. Intra- and interday quantification results are not applicable (n/a) for the patient study. For a full list of assignments and results, see Supporting Information, Tables S7 and S8.

The software-assisted workflow facilitates automatic proteoform assignment by the implementation of a delta mass list within the processing method (Supporting Information, Table S3). For example, the range of possible *N*-glycan structures was previously determined by the bottom-up approach. In addition, cleavage sites and amino acid loss were confirmed via intact protein and middle-up analysis in order to provide a library of potential proteoforms to perform a targeted search against as well as automatic annotation of the masses observed in the intact protein profile. Assignments could then be confirmed using a mass error threshold as well as the expected migration position based on amino acid loss and the number of negatively charged sialic acids present on the proteoform. However, future studies could also focus on confirming glycoform structures directly in the intact protein spectrum by MS/MS experiments, as has previously been shown.^31^

The batch processing of both data sets was enabled by the migration time alignment step which was performed using LaCyTools prior to importing the data into Byos for assignment and quantification. Thus, expected assignments and integration times were verified in a reference file which was then applied to the entire batch of samples. This is illustrated in Supporting Information, Figure S4.A whereby the same integration window (22.09–22.80 min) was used across each sample to integrate and extract deconvoluted spectra of monosialylated species. However, as shown in Supporting Information, Tables S6 and S7, some masses were detected previously by the manual approach, and not by software-assisted annotation. The manual approach utilized smaller integration windows in order to reduce noise within the spectra and perform proteoform annotation followed by manual XIE of the assignments. In this study, integration windows that covered the beginning and end of the XIE peak were used in the software-assisted approach. However, this may also result in the integration of more noise which can affect the mass accuracy for the assignment of some low abundant species (<4%,Supporting Information, Table S6). An example is provided in Supporting Information, Figure S4.B whereby the application of smaller integration windows resulted in the annotation of H5N4F1S1. Despite this, shorter integration windows may also result in greater variability of the extracted deconvoluted spectrum between measurements. Supporting Information, Figure S4.A shows that there are small shifts in the peak apex which will have a greater effect on the deconvolution spectrum when using narrower integration windows. This is further demonstrated in Supporting Information, Figure S4.C whereby integration windows based on integrating the full peak or fwhm of the peak were compared, resulting in average relative standard deviations (RSDs) of 15% and 22%, respectively. Overall, the application of the migration time alignment step and full peak integration improved the throughput and reproducibility of both the data processing and data analysis.

Additionally, quantification via maximum entropy using the DataAnalysis software was compared with the current workflow which employs parsimonious deconvolution. Supporting Information, Figure S5 shows that both approaches resulted in similar relative abundances of selected proteoforms and average RSDs of 21% and 14% for maximum entropy and parsimonious deconvolution, respectively. However, it should be noted that shorter integration windows were used for the maximum entropy approach which may also contribute to the higher RSDs, as previously mentioned. Importantly, a migration time alignment step could not be performed prior to applying the maximum entropy approach due to the software accepting only a single datafile type. Furthermore, integration windows were manually entered in order to extract mass spectra to be used for deconvolution. Thus, although maximum entropy and parsimonious deconvolution gave similar quantification results, the implementation of electropherogram alignment in combination with automatic integration windows resulted in faster and more efficient data processing using the current workflow.

A PSA sequence variant with an additional *N*-glycosylation site was previously reported in urinary PSA from a single patient.^7^ In this study, we explored the data by creating a delta mass list of all possible glycoform combinations based on glycans detected by the bottom-up technique in combination with the altered amino acid sequence whereby Asp_102_ is replaced by Asn. Similar to the approach mentioned above, this allowed us to perform automatic annotation of an additional 12 unique proteoforms (28 in total; Supporting Information, Table S8). However, further validation is required by proteolytic digestion in order to determine the glycans present on the peptide containing the additional *N*-glycosylation site. Additionally, we also observed multiple peaks in the electropherogram of urinary PSA that likely belong to PSA peptides (Supporting Information, Table S5) that may be the result of cocapturing degraded PSA, or the degradation of PSA following capturing.

### Intact Proteoform Quantification

The RSD of the intra- and interday (Table 1) is 12% and 19% by deconvolution as part of the software-assisted workflow, and 11% and 15% by XIE in the manual approach, respectively. The precision determined by both processing methods is within the 20% acceptance criteria applied for other intact protein assays.^32,33^ However, it should be noted that this acceptance criteria generally refers to the absolute quantitation of protein concentration rather than relative abundance. Interestingly, renormalization to the 26 common proteoforms determined by both methods results in intra- and interday RSDs of 9% and 15% (deconvolution), and 12% and 16% (XIE), respectively. Thus, the slightly higher RSDs for the total number of analytes recorded by deconvoluted data processing is likely due to the additional low abundant proteoforms that were detected by this technique. These results are similar to previous studies that determined similar RSDs between both quantification approaches when performing absolute protein quantitation.^11,13,16^ However, Lanshoeft et al. reported that greater precision was achieved when XIC areas were used rather than XIC or deconvoluted peak intensities.^18^ In addition, Kellie et al. demonstrated that protein quantitation was more accurate by the XIC approach at the lower limit of quantification (LLOQ).^17^

**Figure 2:**
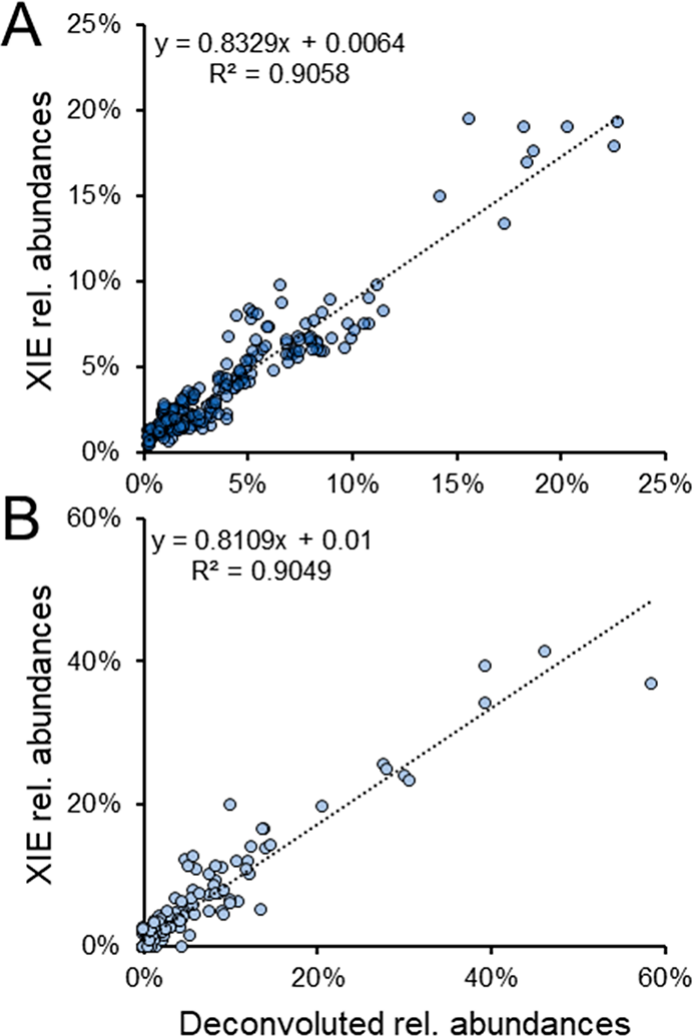
Linear regression plot of relative abundances for common proteoforms quantified by deconvolution and XIE methods. (A) Intra- and interday (*n* = 9) data set. There are 26 common proteoforms detected by both processing methods. (B) Patient (*n* = 8) data set. There are 19 common proteoforms detected by both processing methods. Relative abundances determined by XIE quantification is represented on the *y*-axis and relative abundances determined via deconvoluted quantification is shown on the *x*-axis. The equation of the trendline and *R*^2^ are displayed.

The correlation between the two data processing methods is demonstrated in Figure 2 for the intra- and interday, as well as the patient data sets. This shows the association of the results following renormalization to the common proteoforms determined by both methods. Interestingly, this results in *R*^2^ values of 0.91 and 0.90 for the intra- and interday and patient data sets, respectively. This was also investigated when the most abundant proteoform was omitted from the analysis (Supporting Information, Figure S6), which resulted in *R*^2^ values of 0.83 (intra- and interday) and 0.78 (patients), demonstrating that both processing techniques result in a sufficiently similar quantification of the data. Notably, quantification based on peak intensity has demonstrated greater performance than peak area for the deconvoluted approach.^19^ In the case of quantitative assays using XIEs, peak areas are more commonly reported.^19^ Thus, the use of peak intensities (deconvolution) in comparison with peak areas (XIE) may also introduce some discrepancy between the reported abundance values.

Data processing throughput is an important metric to consider when evaluating new tools for performing intact protein analysis of clinical samples. In this study, we demonstrated the processing of two data sets for which the majority of the data processing steps were software-assisted. For example, the mass calibration and time alignment steps were carried out in half a day while automatic data integration and processing were performed in approximately 1 h for nine samples. Following this, the most abundant mass in each electrophoretic peak was assessed in order to verify the processing had been performed correctly and, as a result of the complex proteoform profiles, the data was exported into a spreadsheet format for further analysis. Thus, the largest hands-on time was due to the data analysis which took approximately 1 day. Overall, the full data processing and analysis was conducted in 2 days for each data set. In contrast, the manual data processing of these data sets was previously performed over several weeks.^7^ In this case, due to the broad XIE peaks as previously mentioned, manual peak integration and deconvolution was performed in order to obtain peak areas and masses, respectively. Thus, this took considerable more hands-on time as this was performed for every analyte in each sample. Furthermore, throughput in terms of proteoform assignment and quantification is an important feature when performing biomarker discovery studies and should be considered when validating methods for intact protein data processing. For example, in this study, the relative abundances of 23 heterogeneous proteoforms were quantified in the patient data set (*n* = 8), resulting in the processing of 127 analytes in total. In comparison, Lanshoeft et al. performed absolute protein quantitation of deglycosylated hIgG1A and [13C]-hIgG1A spiked into rat serum (*n* = 24) resulting in the processing of 48 analytes in total.^18^ Thus, undoubtedly there are different factors to consider when processing clinical samples or biotherapeutics, such as proteoform complexity, and the number of analytes and samples for analysis. In general, these results are similar to previous studies where it was reported that intact data processing could be streamlined via the inclusion of a deconvolution quantification step^19^ and, as a result, similar workflows may be applied in the future for the investigation of larger clinical cohorts using intact protein mass spectrometry.

A one-size-fits-all approach has still not yet been defined for intact protein data processing.^1,12,13,17^ However, several studies prefer the XIC approach^7,9,16,23^ as it remains closer to the raw data and is less prone to the generation of artifacts that may occur due to the inclusion of an extra processing step such as deconvolution.^1,12^ Despite this, as previously mentioned, automation of the majority of the data processing method is required in order to enhance throughput and facilitate the analysis of a greater number of samples. Thus, the XIC approach is currently less amenable to automated processing of intact proteins due to the generation of broad or poorly resolved peaks as a result of overlapping *m*/*z* values in the charge envelopes of different proteoforms. As a result, the extracted ion peak must be manually verified and integrated.^7^ In contrast, only a sufficient mass difference is required in the deconvoluted spectrum in order to perform annotation and quantification simultaneously.^19,20,22^ For example, Wu et al. has recently demonstrated a promising and universally available software that is suitable for identification, deconvoluted quantification, and batch processing of top-down proteomics data.^34^ Thus, deconvolution may be a more suitable approach in order to facilitate greater data processing throughput, and the performance of this method should be further verified in comparison with performing XIC quantification.

Some studies have demonstrated efficient data processing of intact proteins using XICs. For example, Kellie et al. performed deconvoluted mass assignment and XIC quantification in a semiautomated workflow.^9^ However, this approach was applied to proteins up to 10 kDa whereby isotopic resolution was achieved, this resulted in the extraction of specific ion chromatograms. In contrast, such resolution is not commonly observed for proteins that are greater than 25 kDa, resulting in a greater overlap between *m*/*z* signals. This results in the use of the average masses of several charge states, rather than isotopic masses, to generated XICs.^1^ Thus, future work should also focus on the development of such tools which are suitable for larger proteins, as well as enabling a facile user interface that would facilitate greater implementation within the field. Additionally, an improvement in the resolution of larger proteins would also facilitate greater selectivity when performing XIE and XIC quantification.

## Perspectives

The application of vendor-specific software impedes harmonization of practices for intact data processing, particularly in the case of deconvolution as these tools differ in important parameters used for the generation of a deconvoluted spectrum.^12,13,17^ Thus, the development of tools that are capable of handling multiple data formats, such as the data processing steps presented in this study, is an important step toward developing consistent practices in intact protein data processing. In addition, the performance of a *m*/*z*-based migration time alignment, as demonstrated here, may also facilitate automated processing of extracted ion peaks by defining the same integration window in all samples. As a result, in terms of throughput and accuracy, this would enable a fairer comparison between the XIC and deconvolution approaches to be performed.

## Conclusions

In this study, we have built upon our previous work with new data that further support the assignment of cleaved proteoforms in seminal and urinary PSA, including the finding of a potential new cleavage site in seminal PSA. Undoubtedly, a greater understanding of the proteoform profile of PSA from these biological matrices has been achieved which will inform future studies regarding this protein. In addition, we have demonstrated a software-assisted workflow for the annotation and quantification of intact urinary PSA from a small cohort of patients. Importantly, a migration time alignment preprocessing step was performed which allowed the same integration parameters to be used across all samples and, as a result, fast and efficient quantification via deconvolution was achieved. Moreover, the similarity between our results and the extracted ion quantification method was demonstrated. Overall, this work will support the implementation of intact protein data analysis in the biomarker discovery setting.

## Data Availability

The raw data from the intra- and interday and patient studies is deposited in the MassIVE repository, and may be located using the data set identifier MSV000086699.

## Supporting Information Available

Supporting Information 1: Figure S1. Profile of intact seminal PSA analyzed by CE-ESI-MS. Figure S2. Effect of histidine loss on migration time. Figure S3. MS/MS spectra of diagnostic peptides for cleavage variants in seminal PSA. Figure S4. Impact of integration windows on annotation and quantification via deconvolution. Figure S5. Maximum entropy versus parsimonious deconvolution. Figure S6. Linear regression plots excluding the most abundant proteoform. (PDF)
Supporting Information 2: Table S1. Migration time alignment using LaCyTools. Table S2. Integration windows for deconvoluted peak generation in Byos. Table S3. Delta mass in Byos containing expected glycoforms, and cleavage variants with internal amino acid loss. Table S4. Overview of orthogonal data sets supporting proteoform assignments in PSA. Table S5. Assignment of peptide signals discovered in intact urinary PSA electropherogram(s). Table S6. Comparison of urinary PSA quantification results on intra- and interday data set via deconvolution and XIE. Table S7. Comparison of urinary PSA quantification results on patient data set via deconvolution and XIE. Table S8. Urinary PSA sequence variant [D > N] glycoform assignment. (XLSX)
