# Supplementary material for: Software-Assisted Data Processing Workflow for Intact Glycoprotein Mass Spectrometry
Source: J Proteome Res. 2023 Mar 1;22(4):1367–76. doi: 10.1021/acs.jproteome.2c00762 (PMC10088042; doi:10.1021/acs.jproteome.2c00762)
Supplement: Supplementary file 1 — pr2c00762_si_001.pdf [file pr2c00762_si_001.pdf]

Supporting Information

Software-Assisted Data Processing Workflow for Intact Glycoprotein Mass Spectrometry

Alan B. Moran[1], Elena Dominguez-Vega[1], Manfred Wuhrer[1], Guinevere S.M. Lageveen-Kammeijer[1,2]*

1 Leiden University Medical Center, Center for Proteomics and Metabolomics, 2300 RC Leiden, The Netherlands

2 University of Groningen, Department of Analytical Biochemistry, Groningen Research Institute of Pharmacy, Groningen, The Netherlands

* **Correspondence**: Guinevere S.M. Lageveen-Kammeijer, Leiden University Medical Center, Center for Proteomics and Metabolomics, P.O. Box 9600, 2300 RC Leiden, The Netherlands and University of Groningen, Department of Analytical Biochemistry, Groningen Research Institute of Pharmacy, 9713 AV Groningen, The Netherlands; g.s.m.kammeijer@lumc.nl; g.s.m.kammeijer@rug.nl

Table of Contents

Supporting Information 1

Supporting Information – Figures 2

Figure S1. Profile of intact seminal PSA analyzed by CE-ESI-MS 2

Figure S2. Effect of histidine loss on migration time 3

Figure S3. MS/MS spectra of diagnostic peptides for cleavage variants in seminal PSA 4

Figure S4. Impact of integration windows on annotation and quantification via deconvolution 5

Figure S5. Maximum entropy *versus* parsimonious deconvolution 6

Figure S6. Linear regression plots excluding the most abundant proteoform. 7

Supporting Information – Figures

A

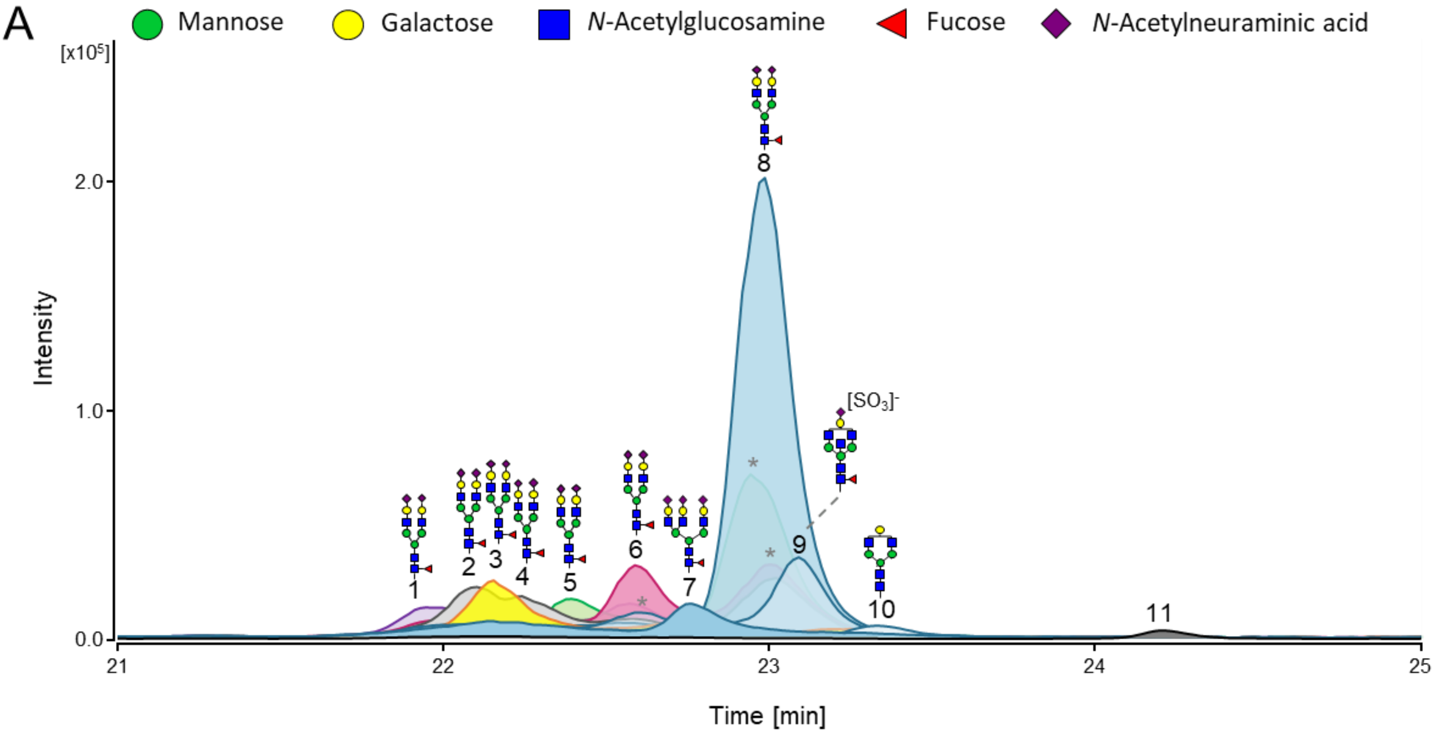

B

	#	#C	C	-AA
purple	**1**	**2**	**K_{169}, K_{206}**	**K**
grey	**2**	**1**	**R_{109}**	**R**
yellow	**3**	**1**	**R_{77}**	**RHSLF**
grey	**4**	**1**	**R_{77}**	**R**
green	**5**	**1**	**K_{169}**	**-**
pink	**6**	**1**	**K_{206}**	**K**
blue	**7 – 10**	**0**	**-**	**-**
black	**11**	**n/a**	**n/a**	**n/a**

Figure S1. Profile of intact seminal PSA analyzed by CE-ESI-MS. (A) XIEs are depicted for representative proteoforms. The most abundant glycoforms are demonstrated whereby the glycan is represented above each peak. Asterisk (*) denotes XIEs from overlapping *m/z* that are present in the charge envelopes of different proteoforms. **(B)** Information regarding each proteoform is provided based on the peak number (#). Abbreviations: “#C”, Number of cleavages; “C”, Cleavage site; “AA”, Observed amino acid loss; “n/a”, Not applicable. In peak 11 *m/z* 1345.05 $[M+8H]^{8+}$ was observed which has a monoisotopic mass of 10752.30 Da.

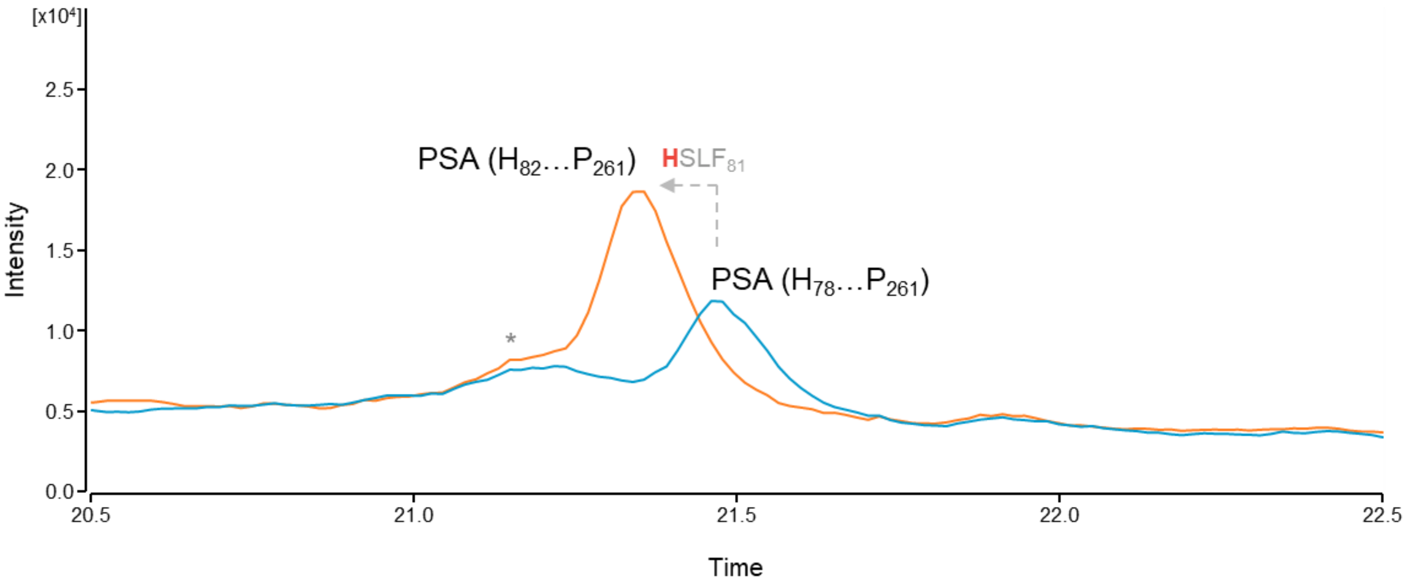

Figure S2. Effect of histidine loss on migration time. The profile of reduced seminal PSA is shown (20.5 – 22.5 min) and the fragments of H_{82}…P_{261} (19804.1 Da) and H_{78}…P_{261} (20289.0 Da) are displayed. H_{78}…P_{261} contains HSLF, however following loss of these amino acids, including the positively charged histidine (marked in red), a decrease in migration time is observed. Asterisk (*) denotes overlapping *m/z* from another proteoform.

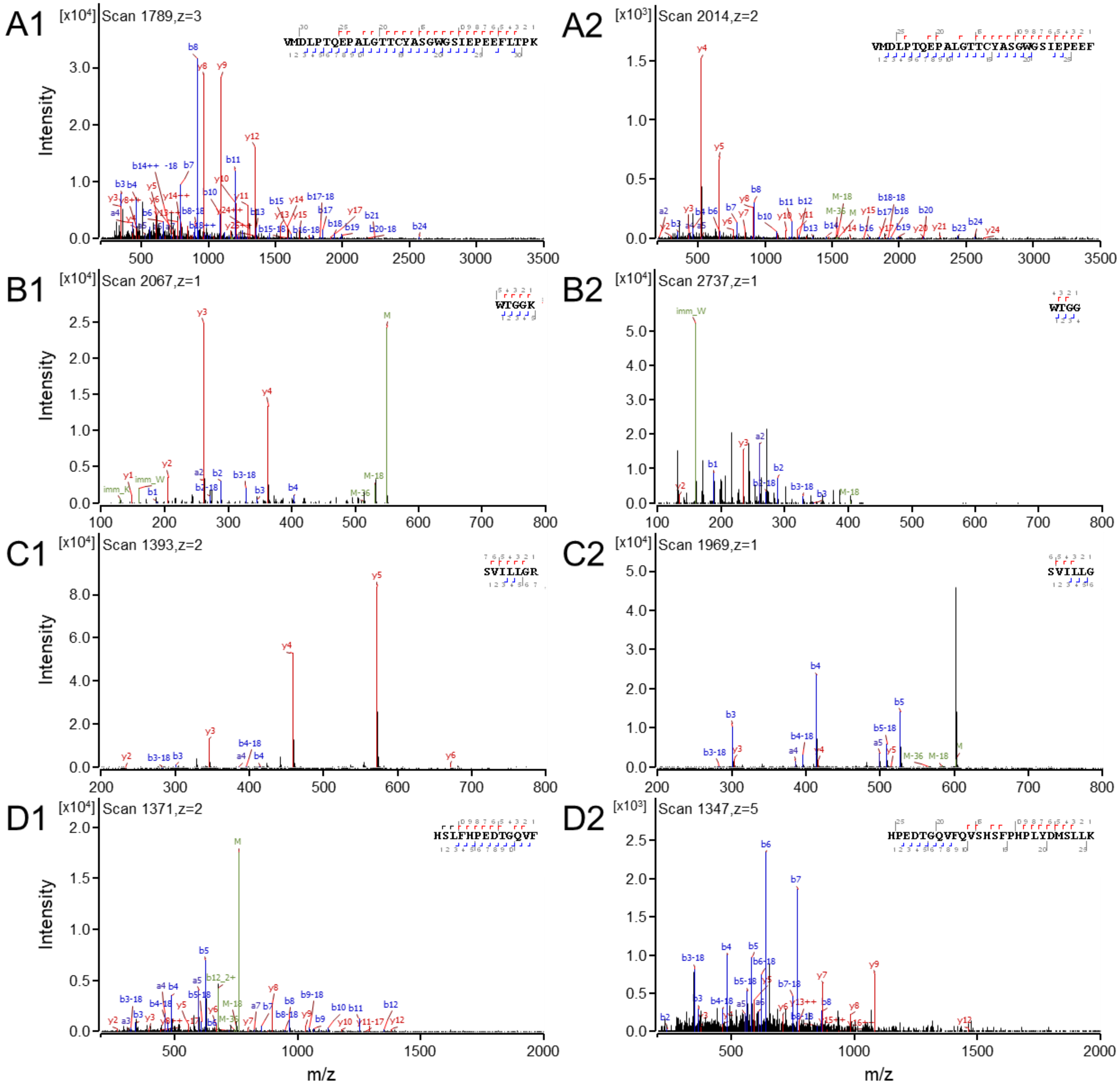

Figure S3. MS/MS spectra of diagnostic peptides for cleavage variants in seminal PSA. Column 1 and 2 show the peptide with no amino acid loss and amino acid loss, respectively, at the cleavage site. **Row A** shows the N-terminal fragment from the cleavage site K_{169} and the loss of LTPK (**A2**). **Row B** shows the N-terminal fragment from the cleavage site K_{206} and the loss of K (**B2**). **Row C** shows the N-terminal fragment from the cleavage site R_{77} and the loss of R (**C2**). **Row D** shows the C-terminal fragment from the cleavage site R_{77} and the loss of HSLF (**D2**). Notably the full tryptic peptide was not found in **D1** which likely shows a degraded peptide that covers the amino acid sequence of HSLF.

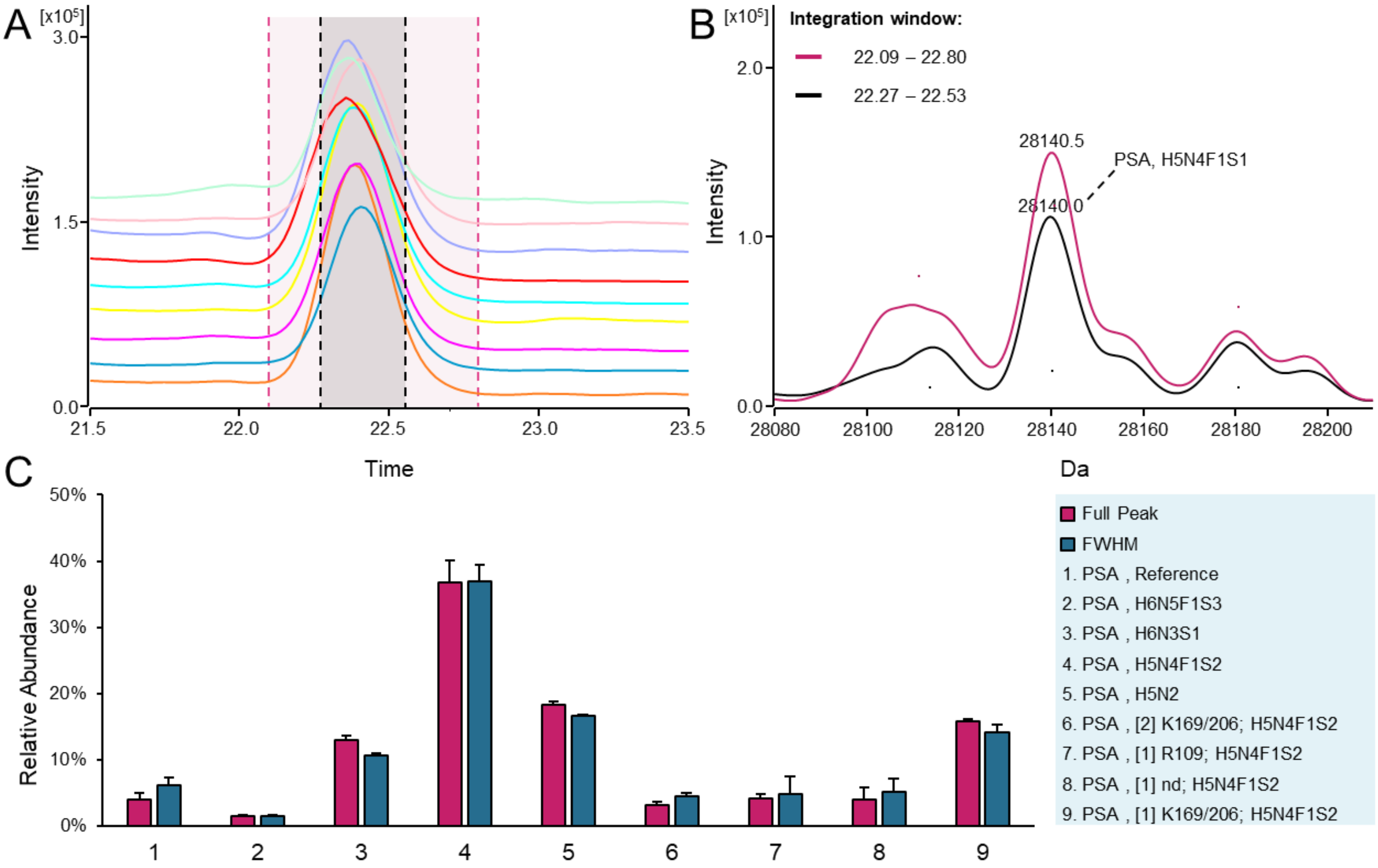

Figure S4. Impact of integration windows on annotation and quantification via deconvolution. (A) XIEs of the most abundant mono-sialylated glycan in urinary PSA, H6N3S1, from the intra- and interday study (*n* = 9). The dotted lines represent the different integration windows, including full peak integration (pink, 22.09 – 22.80 min) and FWHM integration (black, 22.27 – 22.53 min), used to produce deconvoluted spectra. **(B)** An example is provided of the annotation of PSA, H5N4F1S1 across the different integration windows. It may be observed that the full peak (pink) integration window results in the greatest intensity of the deconvoluted peak. The FWHM (black) integration window achieves the assignment PSA, H5N4F1S1 (28140.0 Da, -12.4 ppm) whereas the largest integration window does not (28140.5 Da, -30.2 ppm) when a ± 25 ppm mass error threshold is applied. **(C)** The average relative abundance of the most abundant proteoforms present in each electrophoretic peak, normalized to the total sum of the displayed proteoforms. Error bars represent the standard deviation (*n* = 3). The average RSD for full peak and FWHM integration was 15% and 22%, respectively. The legend is provided in the blue box whereby assignments are displayed in the following format: protein, [number of cleavages], amino acid loss, glycan.

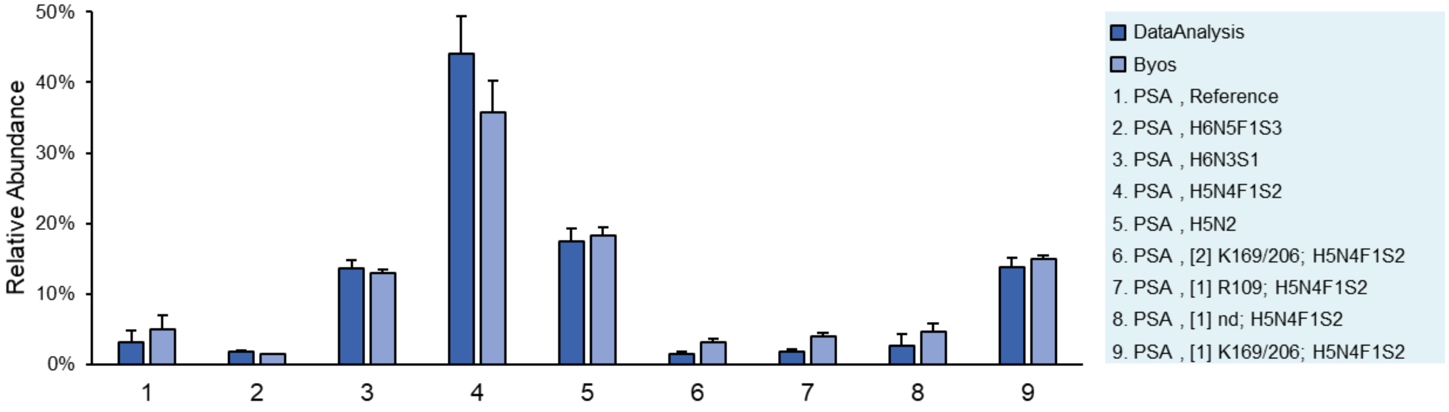

Figure S5. Maximum entropy *versus* parsimonious deconvolution. Maximum entropy deconvolution was carried out using DataAnalysis software whereas parsimonious deconvolution was performed using Byos. The average relative abundance of the most abundant proteoforms present in each electrophoretic peak, normalized to the total sum of the displayed proteoforms is displayed. Error bars represent the standard deviation (n = 3). The average RSD for maximum entropy and parsimonious deconvolution was 21% and 14%, respectively. The legend is provided in the blue box whereby assignments are displayed in the following format: protein, [number of cleavages], amino acid loss, glycan.

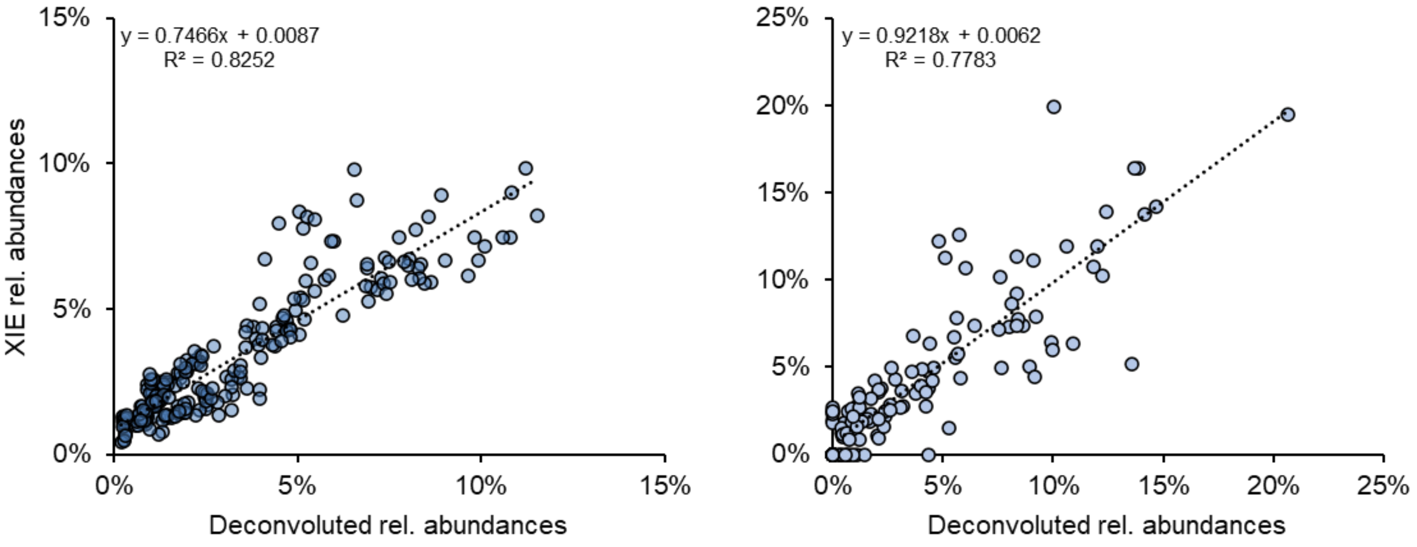

Figure S6. Linear regression plots excluding the most abundant proteoform. (A) The intra- and interday (n = 9) dataset is shown. **(B)** The comparison of the patient dataset (n = 8) is illustrated. Relative abundances determined by XIE quantification is represented on the y-axis and relative abundances determined *via* deconvoluted quantification is shown on the x-axis. The equation of the trendline and R^2 are displayed.
